# How Do the Functional Resemblance Structure and Its Component‐Dependence Change Among the Successional Stages in Degraded Karst Forests?

**DOI:** 10.1002/ece3.72567

**Published:** 2025-11-28

**Authors:** Rui Yang, Qianfei Zhang, Lipeng Zang, Guangqi Zhang, Qingfu Liu, Danmei Chen, Mingzhen Sui

**Affiliations:** ^1^ College of Forestry Guizhou University Guiyang China; ^2^ Guizhou Libo Karst Forest Ecosystem Observation and Research Station Libo China

**Keywords:** functional resemblance structure, karst forests, plant functional traits, succession

## Abstract

The functional resemblance structure, which mainly involved the taxonomic and functional β‐diversity, plays a key role in understanding the assembly process during succession in heterogeneous ecosystems. Systematically comparing the functional resemblance structure is essential for uncovering the successional mechanisms since taxonomic and functional β‐diversity co‐vary. In this study, a series of plots were established among the successional stages in a heterogeneous karst forest. The functional resemblance structure was quantified based on the measured plant functional traits to synchronously compare the changes in taxonomic and functional β‐diversity among the successional stages. The results showed that the taxonomic and functional β‐diversity varied asynchronously across the successional stages, reflecting the changes in functional beta redundancy. In addition, the functional local contribution to β‐diversity was higher in the early successional stage. The functional resemblance structure was dominated by functional beta redundancy, with functioning resilience showing an increasing trend during succession, even in the extremely sensitive karst forests. Furthermore, soil properties mainly determined taxonomic β‐diversity while topography primarily affected the functional dimension. Our findings highlighted the importance of functional beta redundancy in determining the functional resemblance structure and emphasized the necessity of synchronous comparison components of the functional resemblance structure when performing the β‐diversity analyses.

## Graphical abstract

This study was conducted in an extremely heterogeneous karst forest ecosystem. We found asynchronous changes in taxonomic and functional dissimilarities along the succession pathway and the functional redundancy dominated the functional resemblance structure.

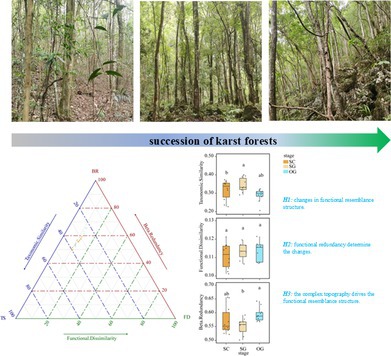

## 1 Introduction

Revealing the ecological processes under the β‐diversity has long been considered an essential approach to understanding the mechanisms of biodiversity maintenance (Bacaro et al. 2007; Legendre and De Cáceres 2013; Podani et al. 2013; Ricotta et al. 2020; Ricotta and Pavoine 2024). However, traditional β‐diversity assessments mainly focused on taxonomic β‐diversity (reviewed by Legendre and De Cáceres (2013), Chao and Ricotta (2019) or Podani et al. (2013)), which might overlook the mechanistic link between species dissimilarity and ecosystem functioning (Lengyel and Botta‐Dukát 2023; Poorter et al. 2021; Ricotta et al. 2020). The functional resemblance structure, which involved both the taxonomic and functional dissimilarities, has been proposed as an essential approach to systematically compare different dimensions of β‐diversity among groups of sites (Poorter et al. 2023; Ricotta et al. 2020; Ricotta and Pavoine 2024). However, fewer studies were conducted on it during succession, where the diversity pattern and its underlying ecological processes varied significantly.

Evidence suggested that taxonomic and functional β‐diversity might decouple during succession (E‐Vojtkó et al. 2023; Ricotta et al. 2020). Firstly, the stronger habitat filtering under harsh conditions in the early successional stage (e.g., nutrient‐poor soils) might reduce functional divergence, lowering functional β‐diversity (Bhaskar et al. 2014; Nakamura et al. 2020; Senior et al. 2013). For another, the taxonomic β‐diversity might increase due to stronger species‐specific colonization at various microhabitats (Bar‐Massada et al. 2014; Dufour et al. 2006; Holl et al. 2013; Jamoneau et al. 2012). Conversely, the stronger limiting similarity at the late successional stage could expand the functional space, thus increasing the functional β‐diversity, even as taxonomic turnover stabilizes (Luo et al. 2021; Schwilk and Ackerly 2005; Violle et al. 2011). These asynchronous responses of taxonomic and functional β‐diversity might induce functional beta redundancy changes (Ricotta et al. 2021; Ricotta and Pavoine 2024). Recent evidence suggested that functional beta redundancy often peaked at the mid‐successional stage, balancing stochastic colonization and deterministic competition (de Bello et al. 2021; Mayfield and Levine 2010; Poorter et al. 2024). Functional β‐diversity variations are determined by the ecological processes dominating the community assembly and can reflect community resilience during succession (E‐Vojtkó et al. 2023; Pillar and Duarte 2010; Poorter et al. 2021, 2024). Thus, comparing the functional resemblance structure and its component dependence is critical for better understanding the mechanisms underlying succession, especially in those heterogeneous conditions with obvious dissimilarity among sites.

Ecologists have invested continuous efforts into quantifying β‐diversity (Baselga 2010; Bernard‐Verdier et al. 2013; Chao and Ricotta 2019; Chiu et al. 2013; Graham and Fine 2008; Grman and Brudvig 2014; Ricotta et al. 2020). By assuming all species are equal, Legendre and Legendre (1998) used the Bray–Curtis coefficient to quantify taxonomic β‐diversity between every two sites based on species abundance data, neglecting the ecological or functional differences among species. Normally, distinct species are functionally dissimilar, which is significantly associated with ecosystem functioning (Castillo‐Figueroa et al. 2025). Podani and Schmera (2021) pointed out that all measurements of distances, dissimilarities, similarities, correlation, association, or proximity among the study objects could be referred to as the generic concept of β‐diversity. Based on this, Ricotta and Pavoine (2024) proposed a statistical framework decomposing the functional resemblance structure into three additive components, including functional dissimilarity, functional beta redundancy, and taxonomic similarity. Firstly, they assumed that all species were equally and maximally distinct. Specifically, the taxonomic dissimilarity between every two sites could be decomposed into two complementary functional components: the functional dissimilarity/similarity among individuals of the species that differed between sites. The functional similarity (i.e., functional beta redundancy) could represent the degree to which the individuals of species not shared by the sites support the same functions (reviewed by Ricotta et al. (2021)). That is, the standardized two functional components (decomposed by taxonomic dissimilarity) and the taxonomic similarity should sum up to1, which could be displayed in a single ternary diagram (Ricotta and Pavoine 2024). Therefore, a comparison of the locations of sites in the ternary diagram could accurately identify the difference in functional resemblance structure among groups of sites, as well as the contributions of the three components to it.

Karst ecosystems often host the most ecologically sensitive vegetation and large species dissimilarity among sites due to their highly heterogeneous bedrock geology and fragile edaphic conditions (Cai et al. 2025; Li et al. 2021; Wu, Yang, Chen, et al. 2024). Despite their roles as unique biodiversity hotspots, karst landscapes, covering approximately 15% of the Earth's surface, often suffer severe degradation under anthropogenic disturbances (Chen et al. 2024; Wang et al. 2019; Wu, Yang, Chen, Sui, et al. 2024). After the disturbances ceased, the degraded karst forests underwent a unique successional pathway to recover a stable community characterized by subtropical karst evergreen‐deciduous mixed forests (Liu et al. 2024, 2021; Peng et al. 2020; Wang et al. 2019). Studies have suggested that the extremely heterogeneous microhabitats in karst landscapes significantly alter the species composition and functional structure within or among successional stages (Chen et al. 2024; Wu, Yang, Chen, Chen, et al. 2024). Studying the biodiversity patterns of degraded karst forests could effectively extend our understanding of successional mechanisms while contributing to regional ecological management (Alroy 2017; Benayas et al. 2009; Cardinale et al. 2012; D'Ettorre et al. 2024). The last decades have seen extensive research on species diversity variations among the successional stages in degraded karst forests (reviewed by Wang et al. (2019) and Wu, Yang, Chen, et al. (2024)). However, most studies focused on species α‐diversity (e.g., species richness or evenness), a critical aspect for revealing underlying ecological processes (Wu, Yang, Chen, Chen, et al. 2024; Wu, Yang, Chen, Sui, et al. 2024). No research has systematically compared the functional resemblance structure and its driving mechanisms during succession, which limits our understanding of the successional mechanism under heterogeneous conditions (Castillo‐Figueroa et al. 2025). Thus, we established a series of plots among the successional stages in a degraded karst forest and measured the plant functional traits. Based on the statistical approach proposed by Ricotta and Pavoine (2024) decomposing the functional resemblance structure, and performing regression analyses, we aim to verify the following hypotheses: (1) the functional resemblance structure changes among the successional stages in degraded karst forests; (2) the functional beta redundancy dominates the changes in the functional resemblance structure across the successional stages; (3) the complex topography of karst landscapes determines the changes in functional resemblance structure.

## 2 Methods and Materials

### 2.1 Study Area and Plot Establishment

The investigation was conducted in Maolan Natural Reserve (25°09′20″–25°20′50″ N, 107°52′10″–108°45′40″ E), southwest China, with annual temperature and precipitation of 18.3°C and 1269 mm, respectively. The region has significant wet and dry seasons, with 86% of the precipitation concentrated from May to October. More particularly, its unique vegetation type of subtropical karst evergreen‐deciduous mixed broadleaved forests is attributed to the heterogeneous habitats and poor soil conditions and dominated by deciduous species ( *Platycarya strobilacea* , *Carpinus pubescens*, and *Pteroceltis tatarinowii*) and evergreen species (*Acer wangchii*, *Boniodendron minus*, and *Clausena dunniana*) (Fu et al. 2019). Due to the poor economic conditions, the forests within the natural reserve experienced various extents of degradation under disturbance (Wang et al. 2019; Wu, Yang, Chen, Chen, et al. 2024). The downhill and marshland were farmed, while the forests uphill were disturbed by grazing. Among the various disturbance regimes, the random selective cutting for firewood was the most prevalent within the reserve (Yang et al. 2024). Following the establishment of the natural reserve, the human inhabitants were resettled elsewhere, and the disturbance ceased. Unlike the subtropical evergreen broad‐leaf forests at similar latitude, the degraded karst forest underwent a unique successional pathway, ultimately transitioning to a stable community driven by soil and topography conditions (Chen et al. 2024; Fu et al. 2019).

**FIGURE 1:**
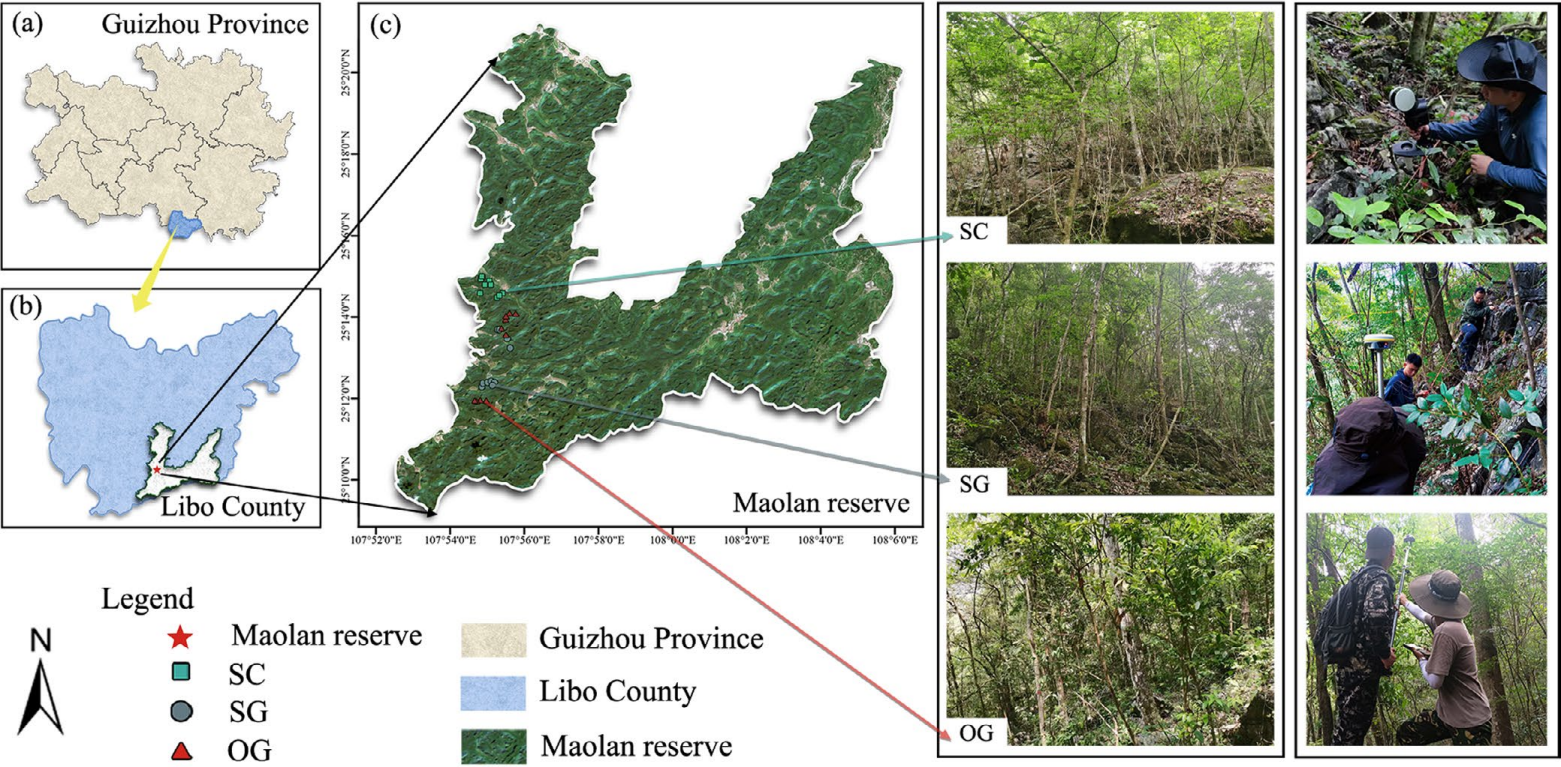
Locations of the plots we established in the study.

Based on the disturbance history and successional pathway, this study considered the shrub‐canopy forest (SC) as the early successional stage and the pioneer canopy forest (SG) as the middle successional stage. The old‐growth forests (OG), undisturbed according to historical records, were considered as the late successional stage (Chen et al. 2024; Wu, Yang, Chen, Chen, et al. 2024; Yang et al. 2024). Following the manual for establishing plots (Condit 1998), ten 30 m × 30 m plots were established in each successional stage from September to November 2021 (*locations of the plots detailed in* Table S1 and Figure 1). Using the differential GPS real‐time kinematic technology (RTK; Galaxy G3, SOUTH, China), we determined the locations of the plots and the 10 m × 10 m grid corners. Within each plot, all the woody individuals with a diameter at breast height ≥ 1 cm were tagged, located, measured for diameter at breast height and height, and subjected to species identification based on the morphological features (*the basic information including density, dominant species, species composition could be seen in* Supporting Informations Tables S1, S2 and Figure S1).

### 2.2 Data Collections

#### 2.2.1 Soil Physicochemical Properties Collection and Determination

Using the five‐point soil sampling method, soil samples were collected to determine the soil physicochemical properties in each plot. A total of 14 indicators were measured, including soil pH, total carbon content (TC), soil organic carbon content (SOC), soil dissolved organic carbon content (DOC), soil total calcium content (Ca), soil total phosphorus content (TP), soil available phosphorus content (AP), soil total potassium content (TK), soil available potassium content (AK), soil total nitrogen content (TN), soil organic nitrogen content (SON), soil available nitrogen content (AN) soil nitrate nitrogen content (NH₄^+^‐N), and soil alkaline hydrolyzable nitrogen content (NO₃^−^‐N). Soil pH was measured using a soil suspension‐pH meter (PH500T; INESA, China). TC and TN were determined using elemental analyzers (UNICUBE trace, Element, Germany). TP was measured using the molybdenum–antimony antispectrophotometric method. TK was detected using inductively coupled plasma‐atomic emission spectrometry (ICPS‐7500; Shimadzu, Japan). SOC was determined using the potassium dichromate oxidation method. DOC was measured after extraction with 0.5 M K_2_SO_4_ solution. The K_2_S_2_O_8_ oxidation method was used to determine SON in soil leachates filtered through 0.45‐μm mixed‐cellulose filters. AN was measured using the alkaline diffusion method. AP was extracted with a mixed solution of 0.05 M HCl and 0.0125 M H_2_SO_4_ and measured with colorimetry. AK and Ca were measured using an atomic absorption spectrophotometer (TAS‐986; PGENERAL, Beijing, China). NH₄^+^‐N and NO₃^−^‐N were extracted with a 2 M KCl solution and measured with colorimetry. The soil physicochemical property differences across successional stages can be found in Table S3.

#### 2.2.2 Topographic Factors Determination

The topography information was mainly acquired by capturing terrain point cloud data using a handheld LiDAR scanner (SLAM100; Shenzhen Feima Robot Technology). First, the “ground point classification” function in LiDAR360 V6.0 was employed to distinguish ground points from objects within the point cloud data. Subsequently, the “digital elevation model” function generated the Digital Elevation Model raster data based on the extracted ground points. Finally, the topographic elevation, slope, aspect, profile curvature, plan curvature, topographic relief, and terrain ruggedness (Roughness) were quantified based on the Digital Elevation Model raster data in ArcMap 10.8. The basic topography information can be found in Table S4. In addition, we performed the principal component analysis on all the abiotic factors (Supporting Information: Figure S2).

#### 2.2.3 Plant Functional Traits Collection and Measurement

According to standardized plant functional traits measurement handbooks worldwide (Pérez‐Harguindeguy et al. 2013), we systematically collected and measured eight dominant plant functional traits, including specific leaf area (SLA, cm^2^/g), leaf dry matter content (LDMC, g/g), leaf thickness (LT, mm), leaf chlorophyll content (CC, SPAD), leaf carbon content (LCC, mg/g), leaf nitrogen content (LNC, mg/g), leaf phosphorus content (LPC, mg/g), and leaf potassium content (LKC, mg/g). The leaf functional traits were determined by randomly collecting at least 5 leaves from 3 to 5 individuals of each species (all individuals if the total is below 3) in each plot. Leaf area was first measured using a high‐speed photography camera (GK821; Deli, China), and then calculated using ImageJ software (National Institutes of Health, USA, http://imagej.nih.gov/ij) and the “LeafArea” package in R software version 4.2.1 (R Development Core Team 2022). Then the fresh leaves were dried in the oven (YT‐700; YETO, China) at 80°C for 48 h, and the dry weights were weighed using an analytical balance (BSA124S; Sartorius, Germany). The LT was determined using electronic digital vernier calipers. The SLA was calculated by dividing the leaf area by the leaf dry weight, and the LDMC was determined by dividing the leaf dry weight by the fresh weight. The CC was measured using a chlorophyll analyzer (TYS‐B; Top Clous‐agri Technology, China). The composite leaf samples of each species were used to determine the LCC, LNC, LPC, LKC. The LCC and LNC were determined using Elemental Analyzers (UNICUBE trace, Element, Germany). Some leaves were digested with H_2_SO_4_‐HClO_4_ and the LPC was determined spectrophotometrically at 700 nm using a continuous flow automated analyzer (AA3; Bran + Luebbe, Germany). The LKC was determined by inductively coupled plasma‐atomic emission spectroscopy (ICP‐AES, ICAP6300; Thermo Fisher Scientific, USA) after wet digestion with HClO_4_‐HF. The above eight leaf functional traits formed the dataset for subsequent analysis (*the basic plant functional traits information can be found in* Table S5).

### 2.3 Data Analysis

#### 2.3.1 Taxonomic and Functional β‐Diversity Variations Across Successional Stages

The taxonomic and functional β‐diversity was separately compared based on the commonly applied Bray–Curtis and Jaccard indices, which captured the differences in species composition based on species abundance/species presence data and had been extended to trait‐based community dissimilarity (Legendre and De Cáceres 2013; Podani and Schmera 2011). Specifically, the Bray‐Curtis index was calculated using relative abundance data, capturing compositional changes while considering species abundance. Meanwhile, the Jaccard index was computed from binary presence/absence matrices, representing compositional turnover regardless of species abundance (Chao and Ricotta 2019; Pavoine and Ricotta 2021). The functional β‐diversity was evaluated following the quantification framework proposed by Ricotta et al. (2020). First, we standardized (zero mean and unit variance) the species‐level functional traits, including SLA, LDMC, LT, CC, LCC, LNC, LPC, and LKC. We then computed pairwise functional β‐diversity among species using the Bray–Curtis distance in the standardized trait space, yielding a species‐by‐species distance matrix. In addition, the distances were normalized by dividing each pairwise distance by the maximum observed value. The functional β‐diversity measurements of each pair of plots were computed based on this trait distance matrix. Specifically, we adopted a tree‐based framework with a functional dendrogram constructed using the Unweighted Pair Group Method with Arithmetic Mean clustering method, which was used to compute community dissimilarity reflecting trait divergence and species composition (Mouchet et al. 2008; Pavoine and Ricotta 2019; Podani and Schmera 2006). The Unweighted Pair Group Method with Arithmetic Mean statistical framework firstly calculated the functional dissimilarity among species and identified the least distance between species. Secondly, the two species with the least functional dissimilarity were merged as a new group and the distance between the new group and other species was quantified again. By repeating the merge and calculation above to the end of all species to one group, the statistical framework finally formed the functional tree with the dendrogram construct (Ricotta et al. 2020). Following the statistical framework proposed by Ricotta et al. (2020), the functional β‐diversity between every two plots was calculated as the average dissimilarity between functionally defined lineages, weighted by their relative abundances in each plot. The calculations were mainly performed using the “FD” and “cluster” packages in R 4.2.1 (R Development Core Team 2022).

To ensure the normality and homoscedasticity of the diversity indices, we firstly performed the K‐S test in R 4.2.1 (R Development Core Team 2022). Then the one‐way ANOVA combined with LSD multiple comparison analyses was implemented to test the significance of the differences in both taxonomic and functional β‐diversity between any two successional stages.

#### 2.3.2 Variation in Functional Local Contribution to β‐Diversity Across Successional Stages

The β‐diversity uniqueness of the sites is important for understanding local β‐diversity patterns. Thus, we quantified the contribution of each plot to the total functional β‐diversity by following the approach proposed by Nakamura et al. (2020), extending the total functional β‐diversity framework to the functional dimension of the community matrix (Legendre and De Cáceres 2013). The functional local contribution to β‐diversity (XLCBD) was calculated by first standardizing the trait matrices and computing the pairwise functional similarity matrix among species based on Bary–Curtis distance. Then, the trait similarity matrix was used to transform the site‐by‐species abundance matrix into a new site‐by‐trait matrix, following the fuzzy approach proposed by Pillar and Duarte (2010). This transformation effectively weights species contributions to a site based on their functional similarity. The transformed site‐by‐trait matrix was used to calculate squared deviations from trait‐wise means across all sites:

(1) $$s_{\mathrm{ij}}={\left(y_{\mathrm{ij}}-{\bar{y}}_{j}\right)}^{2}$$

where the $s_{\mathrm{ij}}$ represents the squared deviations from trait‐wise mean value and the $y_{\mathrm{ij}}$ is the functional representation of site *i* in trait *j*, and ${\bar{y}}_{j}$ is the mean across all sites.

The sum of squared deviations for each site (i.e., row‐wise sum of *S*) is then divided by the total sum of squares across the entire matrix:

(2) $${\text{XLCBD}}_{i}=\frac{\sum_{j}S_{\mathrm{ij}}}{\sum_{i,j}S_{\mathrm{ij}}}$$

This represents the proportion of total functional β‐diversity attributed to each site. A higher XLCBD indicates greater functional uniqueness within the metacommunity. All XLCBD values range from 0 to 1 and sum up to 1 across all sites, facilitating direct comparison among sites and the identification of functionally unique or redundant communities. In addition, the one‐way ANOVA combined with LSD multiple comparison analysis was conducted to examine the difference in XLCBD among successional stages. The analysis was performed using the “BAT”, “cluster,” and “vegan” packages in software version R4.2.1 (R Development Core Team 2022).

#### 2.3.3 Functional Resemblance Structure Decomposition and Ternary Diagram Visualization

Following the statistical framework proposed by Ricotta and Pavoine (2024), we decomposed the functional resemblance structure into three ecological components: functional dissimilarity, taxonomic similarity, and functional beta redundancy. Assuming all species are maximally distinct, the taxonomic dissimilarity was firstly computed using “vegan” package based on the Bray–Curtis distance on species abundance distribution, and the taxonomic similarity should be 1 minus taxonomic dissimilarity (Rao 1982; Ricotta et al. 2020). Then, following the statistical framework raised by Ricotta and Pavoine (2024), the taxonomic dissimilarity was decomposed into functional dissimilarity and functional beta redundancy components, where functional dissimilarity was assumed to minimize the total trait‐based distance between matched individuals across sites. Species‐level trait distances were calculated based on standardized trait data using Euclidean distance and rescaled to the unit interval (Podani and Schmera 2011). Meanwhile, functional beta redundancy represented the functional similarity among unshared species, which is calculated as:

(3) $$R_{\beta }=\mathrm{DS}-\mathrm{DF}$$

(4) $$R_{\beta }+\mathrm{SS}+\mathrm{DF}=1$$

where DS represents taxonomic dissimilarity and SS indicates the taxonomic similarity. *R*_β_ indicates the functional beta redundancy and DF indicates functional dissimilarity. Thus, the three components allow each plot pair to be uniquely represented as a point in a ternary diagram, whose position reflects the balance among the three additive components.

All computations were implemented, and the ternary diagram was constructed using the “vegan,” “ggtern,” and “ggplot2” packages in R 4.2.1 (R Development Core Team 2022).

#### 2.3.4 Factors Determining Functional Resemblance Structure Changes

The effects of soil properties and topography on functional resemblance structure decomposition were examined using the multiple linear regression model, where the taxonomic and functional β‐diversity were considered as response variables and the soil properties and topography as the explanatory variables. Before constructing the multiple linear regression models, all variables were subjected to logarithmic transformation and z‐score normalization. This facilitated the calculation of coefficients that could be compared within and across models, enabling a more rigorous assessment of the relative importance of the predictors. Then, the “lm” function was employed to construct the multiple linear regression models, and the “dredge” function in the “MuMIn” package was used to perform stepwise backward regression (Bartoń 2022). Specifically, the models were selected based on the AICc criterion (Bartoń 2022). Finally, the “rdacca.hp” was adopted to hierarchically partition the parameters of the optimal model, thus yielding the variance explanation rate for each explanatory variable (Lai et al. 2022). The above analyses were performed in R 4.2.1 (R Development Core Team 2022).

## 3 Results

### 3.1 Taxonomic and Functional β‐Diversity Across Successional Stages of Degraded Karst Forests

**FIGURE 2:**
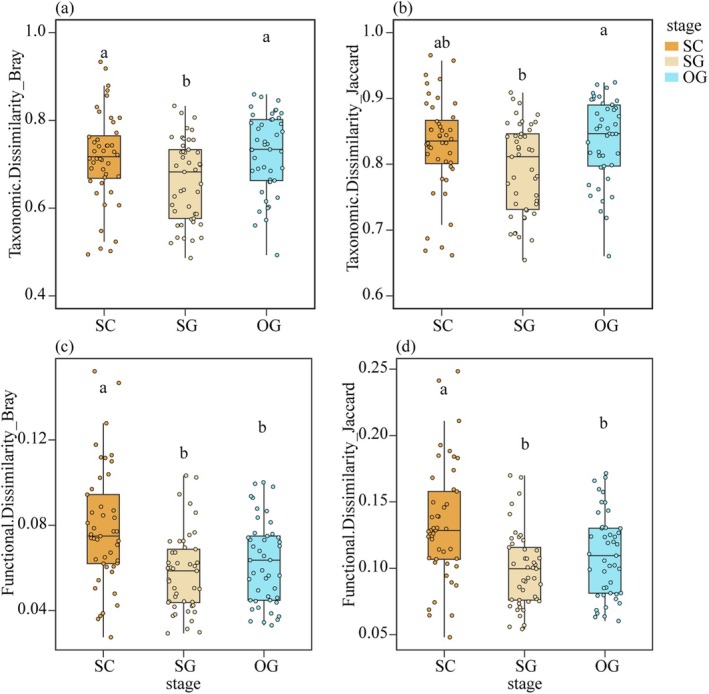
Comparison of taxonomic (a, b) and functional β‐diversity (c, d). Boxes in different colors represent successional stages. SC indicates the early successional stage, SG represents the mid‐successional stage, and OG represents the late successional stage. The significance of the differences is denoted by different letters.

The results showed that the taxonomic β‐diversity based on the Bray–Curtis distance in the middle successional stage was significantly lower than that in the early and late successional stages (Figure 2a). The taxonomic β‐diversity based on the Jaccard distance ranked as the late stage > the early stage > the middle stage (Figure 2b). Unlike taxonomic β‐diversity, functional β‐diversity based on Bray–Curtis and Jaccard distances ranked as the early stage > the middle stage = the late stage (Figure 2c,d). Further analysis showed significant differences in taxonomic and functional β‐diversity across successional stages (Figure S1). Changes in different dimensions of β‐diversity suggested a variation in functional resemblance structure, which supported Hypothesis 1.

### 3.2 XLCBD Variations Across Successional Stages of Degraded Karst Forests

**FIGURE 3:**
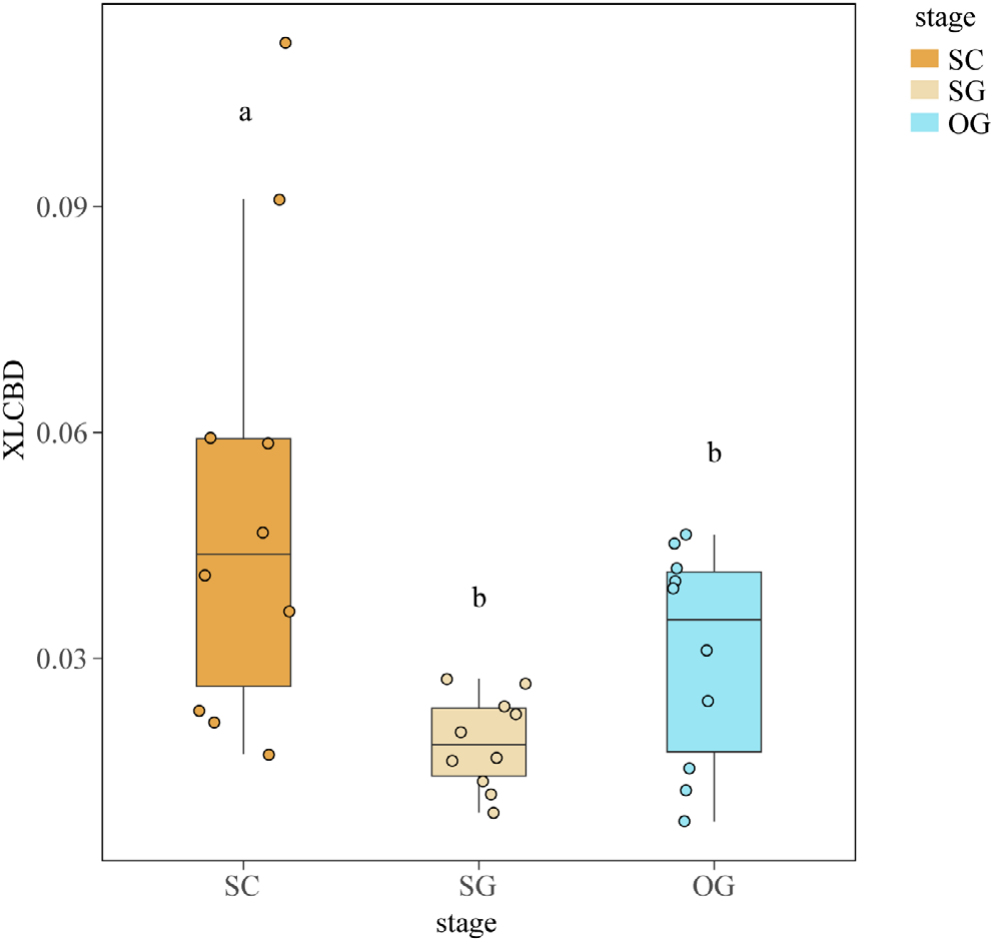
Comparison of XLCBD across successional stages based on Bray–Curtis β‐diversity. Boxes in different colors represent successional stages. SC indicates the early successional stage, SG represents the mid‐successional stage, and OG represents the late successional stage. The significance of the differences is denoted by different letters.

A comparison of XLCBD found that the early successional stage contributed the most. In contrast, the contributions of the middle and late successional stages to the total functional β‐diversity showed no significant difference (Figure 3).

### 3.3 Functional Resemblance Structure Decomposition and Ternary Diagram

**FIGURE 4:**
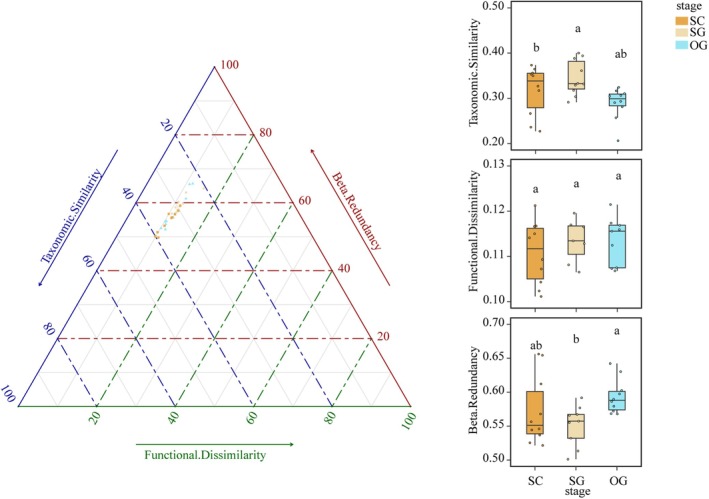
Ternary diagram of the functional resemblance structure across successional stages. Points and boxes in different colors represent successional stages. SC indicates the early successional stage, SG represents the mid‐successional stage, and OG represents the late successional stage. The significance of the differences is denoted by different letters.

All plots were located in the ternary diagram of the functional resemblance structure. The results showed that all plots of the degraded karst forest showed lower functional dissimilarity and were dominated by functional redundancy, which well supported Hypothesis 2. Further analysis showed no significant difference in functional β‐diversity across successional stages (Figure 4). In addition, taxonomic similarity ranked as the middle stage > the late stage > the early stage, and functional beta redundancy ranked the opposite.

### 3.4 Factors Driving Functional Resemblance Structure Variations

**FIGURE 5:**
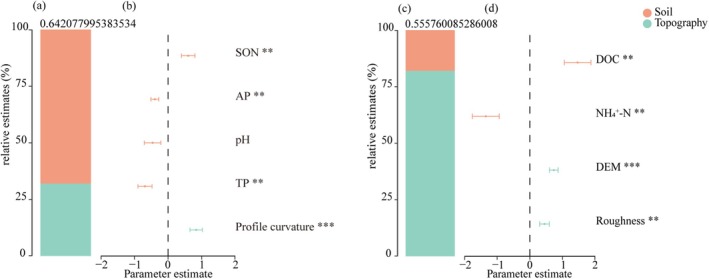
Abiotic factors driving the variations of taxonomic (a, b) and functional β‐diversity (c, d). The column diagrams on the left indicate the contributions of soil properties and topography to β‐diversity. The variables on the right represent the significance of the abiotic factors' contributions to β‐diversity.

The results showed that soil properties primarily influenced taxonomic β‐diversity (Figure 5a), whereas topographic factors mainly determined functional β‐diversity (Figure 5c). Such differential driving mechanisms of taxonomic and functional β‐diversity suggested that the complex topography in karst landscape was not the reason for determining the changes in functional resemblance structure, which did not well support Hypothesis 3. Further analysis showed that the profile curvature, TP, AP, and soil nitrogen were the most important abiotic factors influencing taxonomic β‐diversity among plots (Figure 5b). Meanwhile, roughness, elevation, DOC, and AN mainly affected functional β‐diversity (Figure 5d).

## 4 Discussion

By comparing the functional resemblance structure and its component dependence across successional stages in degraded karst forests, our results accurately distinguished the various changes in taxonomic and functional β‐diversity among the successional stages. These findings highlighted the importance of decomposing the components when performing comparisons on functional resemblance structure, which could well extend our understanding of the successional mechanisms.

### 4.1 Taxonomic and Functional β‐Diversity Changes Among the Successional Stages of Degraded Karst Forests

The variations in species diversity pattern among the successional stages are established, yet the species composition variation pattern is still debated (Chazdon and Brancalion 2019; Poorter et al. 2023; van der Sande et al. 2024). Alexander et al. (2012) suggested that species composition should be dissimilar among sites at the early successional stage due to stochastic dispersal but trend toward similarity with the regional species pool. In contrast, Rozendaal et al. (2019) pointed out that species composition might be similar among sites at the early successional stage since they shared the same regional species pool but could be totally different at the late successional stage. However, Chazdon and Brancalion (2019) pointed out the difficulty of predicting species composition changes due to uncertain mortality and turnover under complex disturbance regimes. Similar to Chazdon and Brancalion (2019), our results also highlighted this uncertainty (Figure 2). Bray–Curtis and Jaccard indices‐based taxonomic dissimilarities tended to decrease first and then increase during succession (Figure 2a,b), with the highest similarity of species composition among plots in the middle successional stage. Consistent with Alexander et al. (2012), the stochastic dispersal might dominate species composition dissimilarity among sites in the early successional stage (Yan et al. 2020). However, the deterministic habitat filtering could severely limit the similar species, thus reducing the taxonomic β‐diversity among sites (van der Sande et al. 2024; Wu, Yang, Chen, Chen, et al. 2024; Yang et al. 2024). Additionally, the heterogeneous microhabitats could exacerbate this trend, with species‐specific colonization at fragmentized patches reducing the competition exclusion, thus contributing to taxonomic β‐diversity (Chen et al. 2024; Wu, Yang, Chen, Sui, et al. 2024).

Unlike taxonomic β‐diversity, functional β‐diversity exhibited a decreasing trend from the early to middle successional stages, and remained stable from the middle to late successional stages (Figure 2c,d). Together with the results of taxonomic β‐diversity, our results supported Hypothesis 1, that the functional resemblance structure showed significant changes across successional stages. Craven et al. (2018) also found a decreasing trend in functional β‐diversity during succession. Ecologists have attributed such patterns to habitat filtering, which severely limits trait convergence, leading to a lower functional β‐diversity among sites within various successional stages (Boukili and Chazdon 2017; Poorter et al. 2021; Segre et al. 2014; Siefert et al. 2013). However, Backhaus et al. (2021) suggested a shift from trait convergence to divergence along the old‐field succession, seemingly contradicting our findings. They argued that the stronger competition limited trait similarity among species. Thus, functional β‐diversity might depend on the relative importance of habitat filtering and limiting similarity to functional traits, resulting in unpredictable functional β‐diversity during succession (Backhaus et al. 2021; Boukili and Chazdon 2017; Siefert et al. 2013; van der Sande et al. 2024). This study found the main difference between functional and taxonomic β‐diversity at the late successional stage, where taxonomic β‐diversity increased while functional β‐diversity remained stable. Purschke et al. (2013) also found that taxonomic β‐diversity increased at the late successional stage, yet most species were likely to be replaced by those with functional similarity, thus increasing the redundancy (Poorter et al. 2024). The functional beta redundancy seemed to determine the changes in functional resemblance structure, which supported Hypothesis 2. Aligned with Siefert et al. (2013), species shared similar functional characteristics despite significant species composition differences among sites, thereby stabilizing ecosystem functioning (Castillo‐Figueroa et al. 2023; Purschke et al. 2013). Such patterns highlighted the importance of assembly processes in determining community structure and maintaining functional resilience during succession. On the other hand, our results showed that the XLCBD of the early successional stage was significantly higher than that of the middle and late successional stages, suggesting a higher proportion of uniqueness in the early successional stage (Figure 3). Previous research found a similar trend in local taxonomic contributions to β‐diversity in karst forests (Wu, Yang, Chen, Chen, et al. 2024). The early successional stage in karst forests seemed to possess unique functional traits (Chen et al. 2024; Meng et al. 2025; Poorter et al. 2021), contributing to the higher β‐diversity in the early successional stage. Interestingly, studies suggested an opposite trend in normal zone, where the functional uniqueness in early successional stage usually showed a relatively lower value compared with that in late successional stages. The extreme microhabitat heterogeneity in karst landscape might contribute to such specialized pattern, where diverse microhabitats allow species with various and unique traits establishing at different local sites (Chen et al. 2024). During succession, taxonomic and functional β‐diversity decreased due to the interspecific competition exclusion. In the stable late successional stage, however, the asynchronous responses of taxonomic and functional β‐diversity might lead to greater ecosystem resilience indicated by functional beta redundancy.

### 4.2 Changes in Functional Resemblance Structure and Potential Driving Mechanisms

Since taxonomic and functional β‐diversity co‐vary during succession, separately comparing the β‐diversity indices might cause misinterpretation of the potential mechanisms (Poorter et al. 2024). Thus, we systematically compared the differences in the functional resemblance structure involving taxonomic similarity, functional dissimilarity, and functional beta redundancy, following the approaches proposed by Ricotta and Pavoine (2024). By simultaneously comparing the functional resemblance structure and its component dependence, we found that the functional beta redundancy dominated the changes in functional resemblance structure across all successional stages in karst forests, which gave clear evidence to support the Hypothesis 2. The functional β‐diversity occupied the lowest proportion and remained unchanged during succession, leading to a contrary trend in taxonomic similarity with functional beta redundancy (Castillo‐Figueroa et al. 2023; Chen et al. 2024). A comparison of the functional resemblance structure also suggested the highest proportion of functional beta redundancy among sites in the late successional stage, consistent with the results above (Figure 3). There findings provided clear evidence that the degraded karst forest showed a higher ecosystem resilience from the perspective of functional traits, seemingly contrary with the findings of Li et al. (2025). The differentiation of ecological assembly processes across spatial scales might be one possible explanation for such results. The severe heterogeneity of karst forests is mainly observed at relatively small spatial scales, resulting in diverging species composition or functional trait patterns within the plots, while species composition seemed to exhibit a converging trend among sites (Wu, Yang, Chen, Chen, et al. 2024; Wu, Yang, Chen, Sui, et al. 2024). Thus, we could observe a higher sensitivity in α‐diversity within plots (Li et al. 2025) and a higher resilience in β‐diversity across plots (Figure 4). Furthermore, the lowest functional beta redundancy in the middle successional stage might mainly result from the highest taxonomic similarity. The stronger competition from the early to middle successional stages replaced the higher proportion of species with lower fitness, allowing the sites to share similar species composition (Ricotta et al. 2020; Ricotta and Pavoine 2024). The decreasing functional space might be the primary reason for the lower functional similarity sharing by species (Pavoine and Ricotta 2019; Ricotta et al. 2021). The taxonomic β‐diversity among sites increased while the functional space remained unchanged from the middle to late successional stages, leading to more species sharing similar functions, thus increasing functional redundancy. Our study highlighted that the functional resilience increased during the succession even in such sensitive and vulnerable karst forest ecosystems.

We initially assumed that the complex topography in karst region should be the determining factor driving the functional resemblance structure. However, results suggested that taxonomic β‐diversity was mainly affected by soil properties, while topographic factors mainly affected functional β‐diversity (Figure 5). Such differential patterns could not well support the Hypothesis 3. The mass‐ratio hypothesis pointed out that the higher nutrients offered advantages in increasing the productivity and species diversity, as well as the taxonomic dissimilarity among sites (Castillo‐Figueroa and Posada 2025; Grime 1998; Liu et al. 2021). Stevens and Carson (2002) also highlighted that resource quantity determined the maintenance of plant diversity rather than resource heterogeneity. specifically, TP and AP played important roles in determining taxonomic β‐diversity (Figure 5b). Research suggested that phosphorus limiting should be the primary limiting factors on the growth, productivity, and recruitment of plants in tropical or subtropical vegetations (Alvarez‐Clare et al. 2013; Castillo‐Figueroa and Posada 2025; Ceulemans et al. 2014; Toro et al. 2023; Ushio et al. 2015). However, Chen, Li, et al. (2025; Chen, Shao, et al. 2025) found that nitrogen limiting might be prevalent in karst forests, which was often ignored in previous studies. Our results also emphasized the importance of soil nitrogen content in modifying the β‐diversity of karst forests (Figure 5b). Considering previous studies (Chen, Li, et al. 2025; Chen, Shao, et al. 2025; Wu, Yang, Chen, Sui, et al. 2024; Yang et al. 2024), the taxonomic β‐diversity of karst forests was limited by soil nitrogen and phosphorus contents. Furthermore, the topographic factors determined the functional dissimilarity among sites in karst forests compared with soil nutrients (Figure 5c), indicating that the extremely heterogeneous microhabitat conditions affected functional β‐diversity. Plants established inn various microhabitats tend to possess unique functional traits, totally different than others across fragmentized microhabitats (Brown et al. 2013; Chen et al. 2024; García‐Palacios et al. 2011). Stronger habitat fragmentation increases functional differentiation by avoiding direct interspecific competition and specific colonization (Bar‐Massada et al. 2014; Stark et al. 2017). Our results suggested that elevation and roughness significantly influenced functional β‐diversity (Figure 5d), emphasizing the importance of small‐scale heterogeneity (within the plot) in driving in functional trait dissimilarity.

The above results (Figure 5) confirmed that the heterogeneous habitat conditions in karst forests mainly affected functional dimension through separate establishment on fragmentized patch of species while soil quality mainly influenced the maintenance of species diversity mainly considering the soil supporting ability. The asynchronous responses of taxonomic and functional β‐diversity to soil nutrients and topography changes determined, at least to some extent, the functioning resilience (functional beta redundancy). Thus, it is essential to simultaneously compare all components of the functional resemblance structure in β‐diversity analyses.

## 5 Conclusions

By systematically comparing the functional resemblance structure and its component dependence among successional stages in karst forests, our study highlighted the functional beta redundancy in determining the functional resemblance structure, even in such sensitive and vulnerable karst forests. In addition, this study suggested differential response of taxonomic and functional β‐diversity to soil nutrient and topography during succession, highlighting that the microhabitat heterogeneity of karst landscape mainly acted on the functional dissimilarity among sites. Our study emphasized the importance of synchronously comparing different dimensions of diversity when performing relative analyses and functional dimension should be paid more attention in biodiversity conservation for heterogeneous karst forests. However, examining the responses of β‐diversity to the heterogeneity of karst forests based on 30 m × 30 m plots might neglect essential scale effects. Future research should involve larger plots and complex habitat gradients, as well as longer temporal monitoring surveys to address these limitations.

## Author Contributions

**Rui Yang:** conceptualization (equal), data curation (equal), formal analysis (equal), funding acquisition (equal), investigation (equal), methodology (equal), validation (equal), visualization (equal), writing – original draft (equal), writing – review and editing (equal). **Qianfei Zhang:** data curation (equal), formal analysis (equal), investigation (equal), methodology (equal), validation (equal), visualization (equal), writing – original draft (equal), writing – review and editing (equal). **Lipeng Zang:** conceptualization (equal), data curation (equal), formal analysis (equal), funding acquisition (equal), investigation (equal), methodology (equal), validation (equal), visualization (equal), writing – original draft (equal), writing – review and editing (equal). **Guangqi Zhang:** funding acquisition (equal), investigation (equal), methodology (equal), writing – original draft (equal). **Qingfu Liu:** conceptualization (equal), data curation (equal), formal analysis (equal), funding acquisition (equal), methodology (equal), resources (equal), supervision (equal), validation (equal), visualization (equal), writing – original draft (equal), writing – review and editing (equal). **Danmei Chen:** funding acquisition (equal), writing – original draft (equal). **Mingzhen Sui:** conceptualization (lead), data curation (lead), formal analysis (equal), funding acquisition (equal), investigation (equal), methodology (equal), validation (equal), visualization (equal), writing – original draft (equal), writing – review and editing (lead).

## Funding

This work was supported by the National Natural Science Foundation of China (32360380, 32360278, 32460377), Science and Technology Program of Guizhou Province (Qian Ke He [2020]1Y011), Forestry Scientific Research Project of Guizhou Province (Qian Lin Ke He [2020]22, [2025]21), Cultivation Project of Guizhou University ([2023]26), and Gui Da Ren Ji He Project ([2021]51).

## Conflicts of Interest

The authors declare no conflicts of interest.

## Supporting information


**Data S1:** ece372567‐sup‐0001‐DataS1.xlsx.

**Table S1:** Locations of the FDPs.
**Table S2:** Important values of dominate species in each successional stage.
**Table S3:** Changes in soil properties along the successional pathway in degraded karst forests.
**Table S4:** Changes in topography along the successional pathway in degraded karst forests.
**Table S5:** Changes in plant functional traits along the successional pathway in degraded karst forests.
**Figure S1:** Dissimilarities in phylogenic (a, b) and functional diversity (c, d) among successional stages in degraded karst forests. Box with different colors indicated successional stages. Results showed a significant difference in phylogenic diversity among successional stage (a, b), as well as similar trends in functional dimension (c, d).
**Figure S2:** PCA of the abiotic factors. The results showed that the profile curvature, TN, pH, acted positively on the PC1, while the DOC and NH_3_^+^‐N contributed most to the negative PC1.

## Data Availability

All the required data are available as Supporting Information.
