# Supplementary material for: How Do the Functional Resemblance Structure and Its Component‐Dependence Change Among the Successional Stages in Degraded Karst Forests?
Source: Ecol Evol. 2025 Nov 28;15(12):e72567. doi: 10.1002/ece3.72567 (PMC12661368; doi:10.1002/ece3.72567)
Supplement: Supplementary file 2 — Table S1: Locations of the FDPs. Table S2: Important values of dominate species in each successional stage. Table S3: Changes in soil properties along the successional pathway in degraded karst forests. Table S4: Changes in topography along the successional pathway in degraded karst forests. Table S5: Changes in plant functional traits along the successional pathway in degraded karst forests. Figure S1: Dissimilarities in phylogenic (a, b) and functional diversity (c, d) among successional stages in degraded karst forests. Box with different colors indicated successional stages. Results showed a significant difference in phylogenic diversity among successional stage (a, b), as well as similar trends in functional dimension (c, d). Figure S2: PCA of the abiotic factors. The results showed that the profile curvature, TN, pH, acted positively on the PC1, while the DOC and NH3 +‐N contributed most to the negative PC1. [file ECE3-15-e72567-s002.docx]

**Supplementary Materials**

**How do the functional resemblance structure and its component-dependence change among the successional stages in degraded karst forests?**

Rui Yang^1^, Qianfei Zhang^1^, Lipeng Zang^1,2^, Guangqi Zhang^1,2^, Qingfu Liu^1,2^, Danmei Chen^1^ AND Mingzhen Sui^1,2*^

^1^ College of Forestry, Guizhou University, Guiyang, 550025, China.

^2^ Guizhou Libo Karst Forest Ecosystem Observation and Research Station, Libo 558400, China.

**Corresponding author:** Mingzhen Sui, College of Forestry, Guizhou University, Guiyang 550025, China.

* E-mail: cafsmz@163.com.

**Supplementary Tables**

**Table S1** Locations of the FDPs

| **FDP** | **Latitude** | **Longitude** | **Density** | **DBH** |
| --- | --- | --- | --- | --- |
| OG1 | N25°13′35.4214″ | E107°55′32.5716″ | 0.5777778 | 5.61±4.56 |
| OG2 | N25°13′46.0541″ | E107°55′24.886″ | 0.31 | 6.86±6.93 |
| OG3 | N25°13′57.5589″ | E107°55′31.1625″ | 0.4866667 | 6.38±5.61 |
| OG4 | N25°14′03.1122″ | E107°55′31.6448″ | 0.2566667 | 7.38±6.62 |
| OG5 | N25°14′06.9275″ | E107°55′47.8362″ | 0.4422222 | 7.31±6.88 |
| OG6 | N25°14′06.9104″ | E107°55′37.0975″ | 0.3066667 | 9.21±7.76 |
| OG7 | N25°11′57.85″ | E107°55′00.5513″ | 0.3277778 | 6.12±7.84 |
| OG8 | N25°11′59.0881″ | E107°54′49.8178″ | 0.2688889 | 7.29±7.27 |
| OG9 | N25°11′57.6824″ | E107°54′44.1513″ | 0.3111111 | 5.76±5.8 |
| OG10 | N25°11′58.6284″ | E107°54′41.3917″ | 0.27 | 6.51±7.46 |
| SG1 | N25°13′19.3901″ | E107°55′38.6261″ | 0.53 | 5.24±5.43 |
| SG2 | N25°13′30.3059″ | E107°55′33.9436″ | 0.5944444 | 4.53±4.59 |
| SG3 | N25°13′44.0249″ | E107°55′23.4246″ | 0.8277778 | 4.97±4.46 |
| SG4 | N25°13′44.3711″ | E107°55′19.9305″ | 0.3766667 | 6.29±6.38 |
| SG5 | N25°12′20.2604″ | E107°54′53.083″ | 0.3522222 | 6.96±7.92 |
| SG6 | N25°12′25.5062″ | E107°54′57.4744″ | 0.3911111 | 6.92±7.66 |
| SG7 | N25°12′25.7197″ | E107°55′02.6021″ | 0.3955556 | 6.43±7.28 |
| SG8 | N25°12′26.8994″ | E107°55′10.1498″ | 0.5444444 | 5±6.23 |
| SG9 | N25°12′25.1896″ | E107°55′12.6783″ | 0.3555556 | 6.03±5.45 |
| SG10 | N25°12′23.2695″ | E107°55′10.9622″ | 0.5366667 | 5.71±5.91 |
| SC1 | N25°14′37.0986″ | E107°54′49.5986″ | 1.0044444 | 3.61±4.22 |
| SC2 | N25°14′50.5256″ | E107°54′57.8418″ | 0.98 | 4.12±3.94 |
| SC3 | N25°14′55.6338″ | E107°55′03.5412″ | 0.78 | 5.22±4.73 |
| SC4 | N25°14′49.9546″ | E107°55′04.8662″ | 0.9788889 | 4.07±3.03 |
| SC5 | N25°15′00.1051″ | E107°54′52.9726″ | 1.24 | 3.66±4.21 |
| SC6 | N25°14′58.412″ | E107°54′50.1893″ | 1.4722222 | 3.8±2.97 |
| SC7 | N25°15′00.9103″ | E107°54′51.9192″ | 0.76 | 4.82±4.74 |
| SC8 | N25°14′30.8336″ | E107°55′16.819″ | 0.6355556 | 4.64±5.15 |
| SC9 | N25°14′36.1918″ | E107°55′28.2332″ | 0.7155556 | 4.58±4.87 |
| SC10 | N25°14′35.1714″ | E107°55′21.322″ | 0.4433333 | 5.58±6.26 |

**Table S2** Important values of dominate species in each successional stage

| SC | | SG | | OG | |
| --- | --- | --- | --- | --- | --- |
| *Ilex triflora* | 0.07314 | *Platycarya strobilacea* | 0.06152 | *Acer wangchii* | 0.05294 |
| *Castanopsis carlesii* | 0.06592 | *Cornus parviflora* | 0.03517 | *Boniodendron minus* | 0.04755 |
| *Loropetalum chinense* | 0.06461 | *Clausena dunniana* | 0.03417 | *Platycarya strobilacea* | 0.04664 |
| *Daphniphyllum oldhamii* | 0.06164 | *Boniodendron minus* | 0.03213 | *Clausena dunniana* | 0.03186 |
| *Symplocos sumuntia* | 0.04249 | *Acer wangchii* | 0.03190 | *Cornus parviflora* | 0.0302 |
| *Lindera communis* | 0.03832 | *Lindera communis* | 0.02909 | *Lindera communis* | 0.02514 |
| *Pinus massoniana* | 0.02963 | *Carpinus pubescens* | 0.02556 | *Carpinus pubescens* | 0.02501 |
| *Castanopsis faberi* | 0.02851 | *Acer coriaceifolium* | 0.02439 | *Handeliodendron bodinieri* | 0.02213 |
| *Myrica rubra* | 0.02570 | *Viburnum henryi* | 0.02345 | *Quercus glauca* | 0.01957 |
| *Lithocarpus glaber* | 0.02069 | *Distylium tsiangii* | 0.0227 | *Pteroceltis tatarinowii* | 0.01928 |

**Table S3** Changes in soil properties along the successional pathway in degraded karst forests

| stage | SC | | SG | | OG | |
| --- | --- | --- | --- | --- | --- | --- |
| SOC (%) | 5.37±0.71b | | 15.17±6.12a | | 15.95±3.19a | |
| TC (%) | 5.28±0.85b | | 14.25±5.19a | | 15.6±3.97a | |
| DOC (mg/kg) | 599.22±43.92a | | 65.13±34.51c | | 149.05±56.06b | |
| Ca (%) | 0.48±0.29b | | 2±0.75a | | 2.39±1.15a | |
| pH | 4.68±0.29b | | 6.78±0.11a | | 6.85±0.15a | |
| TP (mg/g) | 0.22±0.08c | | 3.69±1.18b | | 5.61±2.02a | |
| AP (mg/kg) | 11.13±9.23a | | 16.93±7.35a | | 15.33±6.85a | |
| TK (g/kg) | 0.61±0.29c | | 1.96±0.62b | | 3.35±1.34a | |
| AK (mg/kg) | 131.03±61.07b | | 167.08±51.48ab | | 183.44±45.72a | |
| TN (%) | 0.46±0.1b | | 1.28±0.39a | | 1.42±0.29a | |
| SON (%) | | 0.39±0.09b | | 1.13±0.49a | | 1.27±0.26a |
| NH₄⁺-N (mg/kg) | 77.74±13.58a | | 1.67±0.58b | | 1.37±0.23b | |
| NO₃⁻-N (mg/kg) | 26.14±32.63b | | 56.8±11.56a | | 57.98±31.12a | |
| AN (mg/kg) | 380.52±72.54b | | 536.76±121.93a | | 588.65±71.86a | |

Note: pH is soil pH; TC is soil total carbon content; SOC is soil organic carbon content; DOC is soil dissolved organic carbon content; TN is soil total nitrogen content; SON is soil organic nitrogen content; NH₄⁺-N is soil ammonium nitrogen content; NO₃⁻-N is soil nitrate nitrogen content; AN is soil alkaline hydrolyzable nitrogen content; TP is soil total phosphorus content; AP is soil available phosphorus content; TK is soil total potassium content; AK is soil available potassium content; Ca is soil total calcium content; The significant difference among successional stages was denoted by different letters.

**Table S4** Changes in topography along the successional pathway in degraded karst forests

| stage | SC | SG | OG |
| --- | --- | --- | --- |
| DEM (m) | 819.85±47.93a | 823.71±40.84a | 781.39±28.52b |
| Slope (°) | 20.73±3.29b | 26.62±8.36a | 25.23±5.46ab |
| Aspect (°) | 133.68±65.55b | 224.99±32.88a | 156.41±36.89b |
| Profile curvature (m^-1^) | 87.34±0.54b | 88.28±0.43a | 88.44±0.19a |
| Plan curvature (m^-1^) | 89.15±0.29b | 89.5±0.1a | 89.56±0.07a |
| QFD (m) | 0.11±0.02b | 0.17±0.07a | 0.16±0.05a |
| Roughness (m) | 1.1±0.03b | 1.27±0.16a | 1.23±0.12a |

Note: DEM is Topographic elevation; Slope is Topographic slope; Aspect is Topographic aspect; Profile curvature is Topographic profile curvature; Plan curvature is Topographic plan curvature; QFD is Topographic relief; Roughness is Terrain ruggedness. The significant difference among successional stages was denoted by different letters.

**Table S5** Changes in plant functional traits along the successional pathway in degraded karst forests

| stage | SC | SG | OG |
| --- | --- | --- | --- |
| SLA(cm^2^/g) | 135.19±75.55a | 135.07±59.27a | 141.75±63.71a |
| LDMC(g/g) | 0.43±0.09c | 0.46±0.1a | 0.45±0.1b |
| LT(mm) | 0.16±0.06a | 0.14±0.06b | 0.14±0.06b |
| CC(SPAD) | 44.27±6.08a | 44.17±6.07a | 44.18±6.27a |
| LCC(mg/g) | 447.16±40.87a | 436.66±45.88b | 426.8±39.84c |
| LNC(mg/g) | 15.94±6.14c | 17.72±6.31b | 18.64±6.66a |
| LPC(mg/g) | 1.56±0.23b | 1.62±0.28a | 1.64±0.31a |
| LKC(mg/g) | 12.57±8.1b | 15.41±9.29a | 15.06±8.59a |

Note: SLA is specific leaf area; LDMC is leaf dry matter content; LT is leaf thickness; CC is leaf chlorophyll content; LCC is leaf total carbon content; LNC is leaf total nitrogen content; LPC is leaf total phosphorus content; LKC is leaf total potassium content. The significant difference was denoted by different letters.

**Supplementary Figures**

**
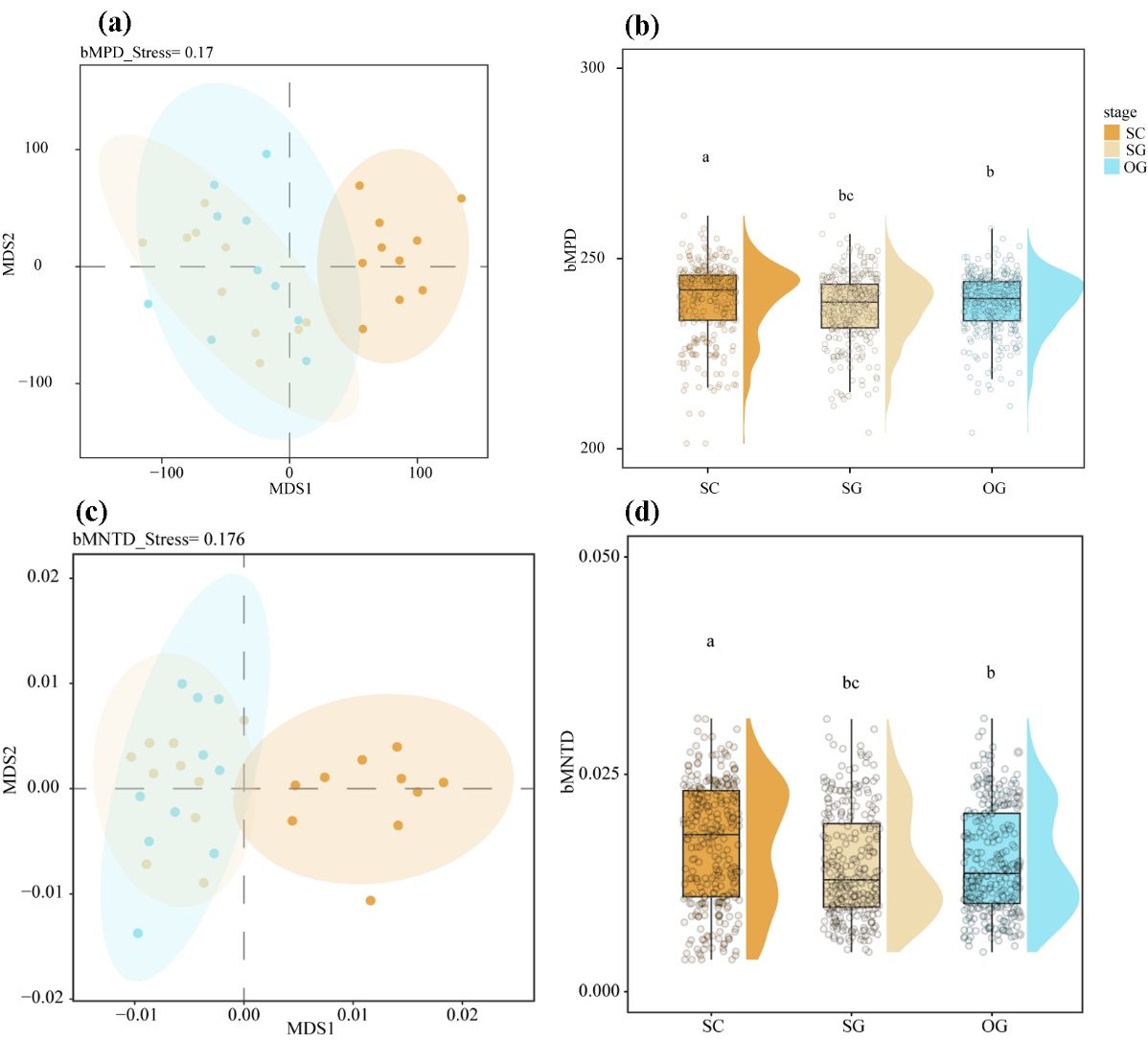
**

**Fig. S1** Dissimilarities in phylogenic (a, b) and functional diversity (c, d) among successional stages in degraded karst forests. Box with different colors indicated successional stages. Results showed a significant difference in phylogenic diversity among successional stage (a, b), as well as similar trends in functional dimension (c, d).


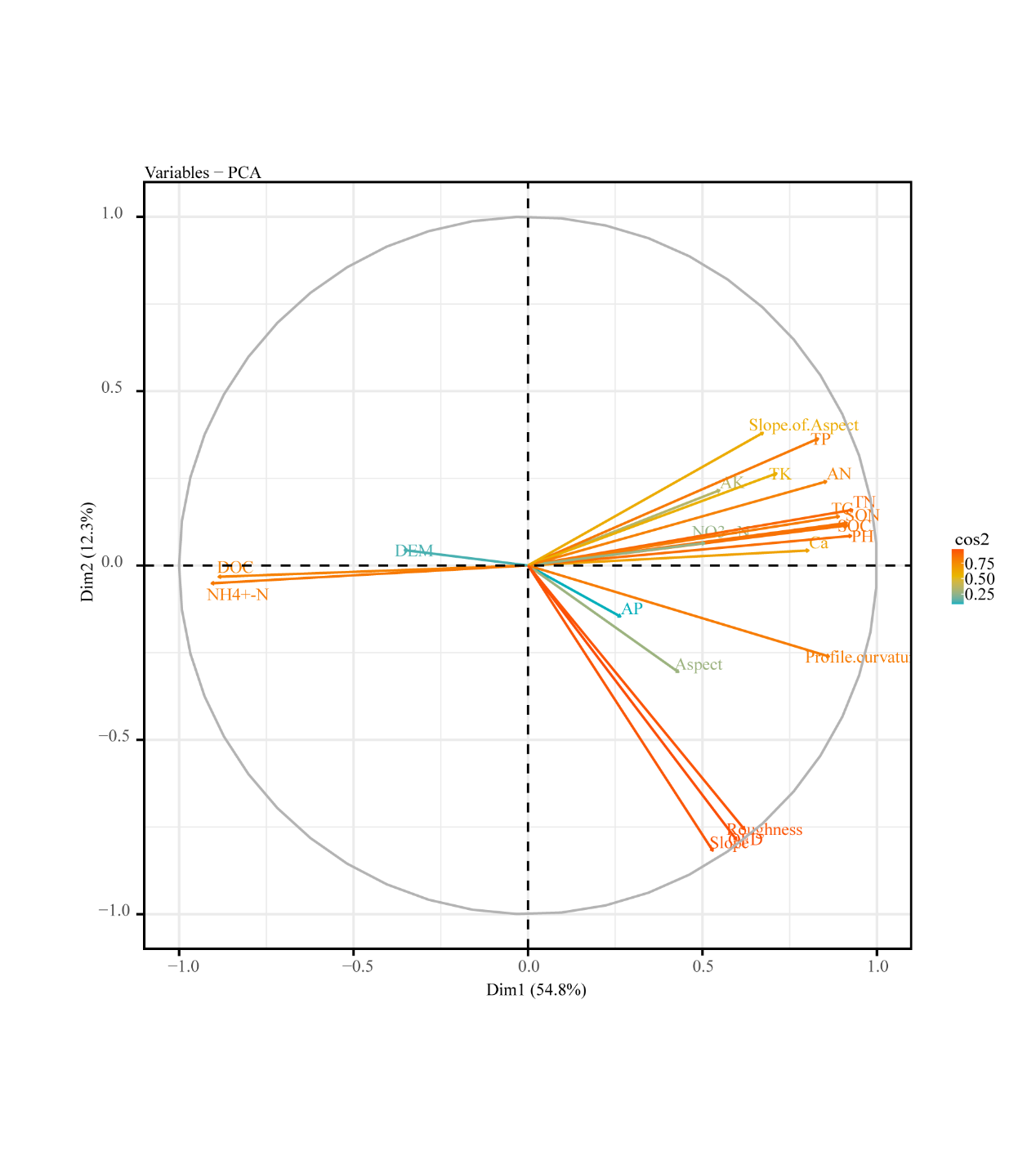


**Fig. S2** PCA of the abiotic factors. The results showed that the profile curvature, TN, pH, acted positively on the PC1, while the DOC and NH_3_^+^-N contributed most to the negative PC1.
